# Dengue Fever Unmasking JAK2 Mutation-Related Polycythemia Vera Presenting With Extensive Splanchnic Venous Thrombosis and Hemorrhagic Complications: A Diagnostic and Therapeutic Challenge

**DOI:** 10.7759/cureus.113352

**Published:** 2026-07-25

**Authors:** Rabbia Ayesha, Fizza Sikandar, Noor ul ain Sikandar, Ayshah Tabasum

**Affiliations:** 1 Department of Internal Medicine, Shifa International Hospital, Faisalabad, PAK

**Keywords:** dengue fever, jak2v617f, mesenteric vein thrombosis, polycythemia vera, splanchnic venous thrombosis, thrombocytopenia

## Abstract

Dengue fever is a common arboviral infection characterized by fever, thrombocytopenia, and hemorrhagic manifestations, whereas thrombotic complications are relatively uncommon. We report a case of severe dengue infection revealing previously undiagnosed *JAK2*-related polycythemia vera (PV) complicated by extensive splanchnic venous thrombosis. A 58-year-old male patient with a history of diabetes mellitus and hypertension presented with a five-day history of fever, anorexia, and abdominal pain. Initial laboratory investigations demonstrated progressive thrombocytopenia, elevated hemoglobin, and increased hematocrit. During hospitalization, platelet counts continued to decline, and dengue serology returned positive. Computed tomography of the abdomen and pelvis revealed extensive splanchnic venous thrombosis involving the superior mesenteric, splenic, and portal venous systems, along with acute pancreatitis. The patient subsequently developed mucosal bleeding. Further evaluation revealed splenomegaly and a positive *JAK2* V617F mutation, confirming underlying polycythemia vera. Due to concurrent bleeding and thrombosis, anticoagulation was initially deferred until hematologic stabilization was achieved. The patient was subsequently treated with anticoagulation and hydroxyurea, resulting in clinical recovery. This case highlights the rare coexistence of hemorrhagic and thrombotic complications in dengue fever and emphasizes the importance of considering underlying myeloproliferative neoplasms in patients presenting with atypical thrombosis, erythrocytosis, or splenomegaly.

## Introduction

Dengue fever is one of the most common arboviral infections worldwide and may present with a spectrum of illness ranging from uncomplicated febrile disease to severe dengue characterized by thrombocytopenia, bleeding, plasma leakage, and organ involvement [1]. Although hemorrhagic manifestations are well recognized, thrombotic complications have increasingly been reported and may be underrecognized in clinical practice [2-4].

Polycythemia vera (PV) is a chronic myeloproliferative neoplasm characterized by erythrocytosis and an increased risk of arterial and venous thrombosis. Splanchnic venous thrombosis may represent the initial manifestation of previously undiagnosed PV, particularly in patients harboring the *JAK2* V617F mutation [5-8].

The coexistence of severe dengue and PV presents a unique therapeutic challenge because clinicians must simultaneously balance competing risks of hemorrhage and thrombosis. This uncommon combination complicates diagnostic evaluation and therapeutic decision-making, with limited evidence available to guide management.

We report a case of severe dengue infection complicated by profound thrombocytopenia, mucosal bleeding, acute pancreatitis, and extensive splanchnic venous thrombosis that ultimately led to the diagnosis of previously unrecognized *JAK2* mutation-related PV.

## Case presentation

A 58-year-old male patient with a known history of diabetes mellitus and hypertension presented with a five-day history of low-grade fever, anorexia, and abdominal pain. He had initially been managed at home with oral rehydration solution and analgesics.

On the sixth day of illness, a complete blood count demonstrated thrombocytopenia with a platelet count of 80,000/µL, hemoglobin of 17.3 g/dL, and elevated hematocrit of 53%, prompting presentation to the emergency department (Table 1). Repeat investigations revealed further decline in platelet count. Ultrasonography of the abdomen demonstrated splenomegaly.

**Table 1:** Serial hematological parameters demonstrating progressive thrombocytopenia, followed by improvement after platelet transfusion. Hb: hemoglobin; HCT: hematocrit; CBC: complete blood count

CBC Timeline	WBC (Reference: 4.0-11.0 (×10⁹/L))	Hb (Reference: 13–17 (g/dL))	HCT (Reference: 40–50 (%))	Platelets ( Reference: 150-450 (×10⁹/L))
Presentation (Day 0)	3.00	17.3	53.0	80
Emergency Department Admission (Day 0)	3.09	16.1	46.2	40
Repeat (Day 1)	3.88	15.2	44.1	36
Nadir (Day 2)	3.51	15.9	45.7	20
After Platelet Transfusion (Day 3)	4.06	15.4	44.2	51

The patient was admitted for further management and received intravenous fluid therapy. Dengue serology was requested. Owing to the elevated hematocrit and splenomegaly, evaluation for an underlying myeloproliferative neoplasm, including *JAK2* mutation testing, was initiated.

Within 48 hours of admission, platelet counts declined further to 20,000/µL. Platelet transfusions were administered. Contrast-enhanced computed tomography (CT) of the abdomen and pelvis demonstrated an eccentric thrombus in the superior mesenteric vein and its distal branches, extending into the portosplenic confluence, the main portal vein, and its anterior branch. Associated radiologic findings were consistent with acute pancreatitis. 

Subsequently, dengue serology returned positive (Table 2). The patient developed mucosal bleeding from the oral cavity in the setting of severe thrombocytopenia. Platelet transfusion, tranexamic acid mouthwash, and broad-spectrum antibiotics were administered. Over the following 24-36 hours, platelet counts gradually improved.

**Table 2:** Key diagnostic investigations supporting dengue infection and underlying polycythemia vera. NS1 antigen positivity confirms acute dengue infection in the appropriate clinical setting. Detection of *JAK2* V617F mutation is strongly associated with myeloproliferative neoplasms and supports the diagnosis of polycythemia vera in this clinical context.

Investigation	Result	Clinical interpretation
Dengue NS1 antigen	Positive	Suggestive of acute dengue virus infection
*JAK2* V617F mutation	Positive	Supporting the diagnosis of an underlying myeloproliferative neoplasm (polycythemia vera)

Once bleeding was controlled and the platelet counts reached 51 × 10⁹/L, anticoagulation was initiated with unfractionated heparin 5,000 units subcutaneously every 12 hours for 48 hours. Thereafter, the patient was transitioned to oral rivaroxaban 20 mg once daily. 

*JAK2* mutation testing subsequently returned positive (Table 2). Based on the initial erythrocytosis, splenomegaly on abdominal ultrasonography, and the presence of a *JAK2* mutation, a diagnosis of PV was established. The patient was subsequently started on hydroxyurea 500 mg twice daily. 

At six-week follow-up, the patient remained clinically stable without recurrent abdominal symptoms. He continued rivaroxaban and hydroxyurea under hematology follow-up.

## Discussion

Dengue fever is traditionally regarded as a hemorrhagic disease because of its association with thrombocytopenia, coagulopathy, and bleeding manifestations [1]. However, thrombotic complications have increasingly been recognized, including deep venous thrombosis, cerebral venous thrombosis, pulmonary embolism, and mesenteric venous thrombosis [2-4]. Our patient presented with severe thrombocytopenia and mucosal bleeding consistent with severe dengue. However, the coexistence of elevated hemoglobin, elevated hematocrit, splenomegaly, and extensive thrombosis involving the mesenteric, splenic, and portal veins suggested an additional underlying prothrombotic condition.

PV is one of the most important causes of splanchnic venous thrombosis. Multiple studies have demonstrated a significant prevalence of *JAK2 *V617F mutations among patients presenting with portal, splenic, and mesenteric venous thrombosis, even in the absence of an established diagnosis of a myeloproliferative neoplasm [6-8]. Furthermore, splanchnic venous thrombosis may represent the initial clinical manifestation of otherwise occult PV [8].

The extensive thrombosis observed in this patient was likely multifactorial, occurring in the setting of acute dengue infection and previously undiagnosed *JAK2*-related PV. Dengue may contribute through endothelial dysfunction, inflammation, hemoconcentration, and activation of the coagulation pathways [2-4], whereas PV provides a chronic prothrombotic state [5].

Anticoagulation is the cornerstone of treatment for mesenteric venous thrombosis [9,10]; however, in our case, management posed a significant challenge as the patient simultaneously exhibited severe thrombocytopenia and active mucosal bleeding. Anticoagulation therefore had to be delayed until hematologic stabilization was achieved. Once platelet counts improved, anticoagulation was successfully initiated without further hemorrhagic complications.

The diagnosis of *JAK2* V617F mutation-positive PV substantially altered long-term management. Current recommendations emphasize thrombosis prevention through hematocrit control and cytoreductive therapy in high-risk patients, with hydroxyurea remaining a commonly utilized first-line cytoreductive agent [5,11].

## Conclusions

This case highlights the rare coexistence of hemorrhagic and thrombotic manifestations in a patient with dengue fever and previously undiagnosed *JAK2* mutation-related PV. The presence of erythrocytosis, splenomegaly, and splanchnic venous thrombosis should prompt evaluation for an underlying myeloproliferative neoplasm, even in the setting of severe thrombocytopenia. Early recognition of this dual pathology is essential because it significantly influences both acute management and long-term prognosis.
